# Charity vs. Policy

**Published:** 1875-05

**Authors:** 


*Charity vs. Policy.*

The exercise of the principle of charity does not, necessarily, imply insensibility to personal injury or indifference to the violation of law. The individual who, by malevolence, has wantonly insulted another, and inflicted serious injury upon his good name and standing among his fellowmen, cannot and ought not to expect to receive the benefit of a charitable, forgiving spirit from the injured party, except the latter be thoroughly convinced that the offender is influenced by sincere contrition for the baseness or injustice of his act, and that he voluntarily desires to make the *amende honorable*, like an honest gentleman.

If his conscience admits, and the law proves, that he has wronged a fellew being, and brought reproach upon truth and honor, it is his duty, if he has any regard for honor and propriety, to make a *frank* confession of his guilt in as public a manner as the offense was made, and to offer such candid confession as a reparation for the injury done, and as a claim to the pardon sought.

Any other than this open and straightforward method cannot be recognized among reliable gentlemen—it is strictly a question of *principle* and *right,* and can never be placed upon grounds of so-called compromise. To compromise is to admit mutual guilt or blame; how, then, can compromise be admitted in a matter, for instance, where one party has been convicted as the offender, and the other is proven to be the innocent and injured party? This is illogical and absurd. Nor should the offender endeavor to lighten the burden of his guilt, or strive to hoodwink his conscience and the public by the plea that others like himself were accessory in inflicting injury upon an innocent man, and that the pardon extended to these minor transgressors is equally applicable to him—the fountain-head of a deep and filthy stream of slander and vituperation. Let every tub stand upon its own bottom.

If you *know* that you have insulted and injured a man, walk up to him *like* a man, acknowledge the misdemeanor in its fullest extent, and ask him to forgive you in as free and frank a spirit as that in which the pardon is asked.

The artifice to circumvent an unpleasant duty, and by a series of "ifs," "and that is," etc., *ad nauseam,* to shirk a plain fact, is as unmanly as it is hypocritical. He and some of his inconsiderate friends may call it "policy;" but it is not the policy of honesty—the only policy honorable men should recognize.

"Pay as you go," is an adage as applicable to our moral duties as it is to the economical management of our financial affairs.

If you have committed an acknowledged offense against the welfare of a community, or to the injury of an individual, *pay the penalty,* and don't expect to receive a full pardon until the law of right and of justice has been fully satisfied in your case. You will then feel that you deserve the benefit of charity, and are entitled to a free pardon.

And, by-the-way, we would observe that no man should consider any one a friend of his who would induce him to pursue a different line of conduct, on the score of "policy." It is like the whispering of Satan in the ears of Eve. Beware of it. No true man, in fact, will need advice as to what to do when duty and honor point the way; he will never attempt to make what he knows to be wrong, to appear to be right.

